# Extracting Instantaneous Respiratory Rate From Multiple Photoplethysmogram Respiratory-Induced Variations

**DOI:** 10.3389/fphys.2018.00948

**Published:** 2018-07-18

**Authors:** Parastoo Dehkordi, Ainara Garde, Behnam Molavi, J. Mark Ansermino, Guy A. Dumont

**Affiliations:** ^1^Electrical and Computer Engineering, Faculty of Applied Science, The University of British Columbia, Vancouver, BC, Canada; ^2^Faculty of Electrical Engineering, Mathematics and Computer Science, University of Twente, Enschede, Netherlands; ^3^Lionsgate Technologies, Vancouver, BC, Canada; ^4^Anesthesiology, Pharmacology and Therapeutics, The University of British Columbia, Vancouver, BC, Canada

**Keywords:** instantaneous respiratory rate, pulse oximetry, photoplethysmogram, respiratory-induced variation, synchrosqueezing transform

## Abstract

In this study, we proposed a novel method for extracting the instantaneous respiratory rate (IRR) from the pulse oximeter photoplethysmogram (PPG). The method was performed in three main steps: (1) a time-frequency transform called synchrosqueezing transform (SST) was used to extract the respiratory-induced intensity, amplitude and frequency variation signals from PPG, (2) the second SST was applied to each extracted respiratory-induced variation signal to estimate the corresponding IRR, and (3) the proposed peak-conditioned fusion method then combined the IRR estimates to calculate the final IRR. We validated the implemented method with capnography and nasal/oral airflow as the reference RR using the limits of agreement (LOA) approach. Compared to simple fusion and single respiratory-induced variation estimations, peak-conditioned fusion shows better performance. It provided a bias of 0.28 bpm with the 95% LOAs ranging from −3.62 to 4.17, validated against capnography and a bias of 0.04 bpm with the 95% LOAs ranging from −5.74 to 5.82, validated against nasal/oral airflow. This algorithm would expand the functionality of a conventional pulse oximetry beyond the measurement of heart rate and oxygen saturation to measure the respiratory rate continuously and instantly.

## 1. Introduction

Respiratory rate (RR), along with other vital signs like heart rate (HR) and blood pressure, is monitored for primary or continuous assessment of patient wellness. There is significant evidence that an abnormal respiratory rate is an important predictor of serious illness. For example, in children aged 1–5 years old, an elevated RR (>40 breaths/min) is an important criterion for the diagnosis of pneumonia (WHO, 2013). Furthermore, Fieselmann et al. analyzed the measurements of vital signs during the 72 h prior to cardiac arrest and showed that a high respiratory rate (>27 breaths/min) was a significant predictor of cardiac arrest in hospitals (Fieselmann et al., 1993). In addition, Subbe et al. showed that relative changes in respiratory rate are much more significant than changes in HR or systolic blood pressure in unstable patients and therefore the respiratory rate is more likely to be a better predictor for identifying the patient at risk (Subbe et al., 2003).

RR can be measured by a nurse counting the number of times the chest rises in 1 min (Lovett et al., 2005). Continuous monitoring of RR, though, needs a monitoring device and can be performed using capnography, transthoracic impedance pneumography, nasal/oral pressure transducers and abdominal/thoracic respiratory inductance plethysmography belts, among others. However, recent studies have found that neither the nurses nor the monitoring devices provide accurate and reliable measurements of RR (Lovett et al., 2005). Therefore, there is a clear need for a robust, automatic, reliable and non-invasive measure of RR for performing a spot-check and for continuous monitoring.

Analysis of the photoplethysmogram (PPG) recorded using a pulse oximeter could offer an alternative method for monitoring RR. The PPG waveform contains information about a wide range of physiological parameters such as HR, heart rate variability (HRV), oxygen saturation (SpO_2_), vascular tone, blood pressure, cardiac output and respiration (Shelley, 2007). However, most conventional pulse oximeters only provide information about HR and SpO_2_. In this study, we have presented an algorithm for robust estimation of instantaneous respiratory rate (IRR) from PPG with the aim of developing a portable solution based on pulse oximetry, suitable for both continuous monitoring and spot-check applications.

### 1.1. Background

A pulse oximeter measures the blood volume changes or PPG, based on the light absorption characteristics of the blood at the measuring site on the body (e.g., finger, forehead, and earlobe). A conventional transmission pulse oximeter sensor consists of two LEDs, red and infra-red, and a photo-detector. The LEDs emit light at both red and infrared wavelengths (650 and 950 nm, respectively) and the photo-detector captures the transmitted light. Based on the Beer-Lambert's law, the transmitted light density decreases during systole when the peripheral arterial blood volume is at its maximum value and increases during diastole. The PPG signal generated in the photo-detector, then, has a pulsatile waveform (AC) whose peaks and troughs reflect light transmitted through the tissue when blood volume is minimal and maximal, respectively. PPG also has a large baseline component (DC) mainly rises because of constant absorption and scattering of light traveling through skin, bones and tissues. The small variation observed in DC is mostly due to venous blood variation which changes the intensity of the light captured by the photo-detector (Webster et al., 1997).

**Figure 1:**
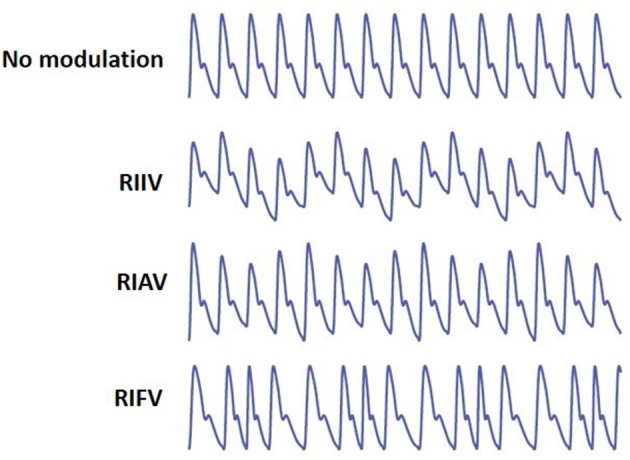
From top, PPG with no modulation, Respiratory-Induced Intensity Variation (RIIV), Respiratory-Induced Amplitude Variation (RIAV), and Respiratory-Induced Frequency Variation (RIFV). Adapted from Addison et al. (2012).

Respiration may induce variation in PPG in three different ways (Meredith et al., 2012) (Figure 1):

1. Respiratory-Induced Intensity Variation (RIIV): Changes in venous return due to changes in intra-thoracic pressure throughout the respiratory cycle cause a baseline (DC) modulation of the PPG signal. During inspiration, decreases in intra-thoracic pressure result in a small decrease in central venous pressure increasing venous return. The opposite occurs during expiration. As the venous bed at probing site cyclically fills and drains, the baseline is modulated accordingly.
2. Respiratory-Induced Amplitude Variation (RIAV): During inspiration, left ventricular stroke volume decreases due to changes in intra-thoracic pressure leading to the decreased pulse amplitude. The opposite happens during expiration.
3. Respiratory-Induced Frequency Variation (RIFV): HR varies throughout the respiratory cycle; HR increases during inspiration and decreases during expiration. This phenomenon well-known as respiratory sinus arrhythmia (RSA) is mainly due to the autonomic regulation of HR during respiration.

Respiration may induce variation in PPG differently among different individuals in health and disease. For instance, RIFV, as an indicator of autonomic activity, maybe affected by diseases and disorders (e.g., myocardial infarction, diabetic neuropathy or sleep breathing disorders Dehkordi et al., 2016). RIAV and RIIV are also very sensitive to dehydration and hypovolemia. In addition, respiratory-induce variations are different for women and men (Li et al., 2010). Estimation of IRR by combining the information from three respiratory-induce waveform variations, then, improves the algorithm performance and increases the robustness of results (Karlen et al., 2013).

Many algorithms have been proposed to estimate RR from PPG. Auto-regression (Thayer et al., 2002), Fourier transform analysis (Karlen et al., 2013), correntropy spectral density (Garde et al., 2014), digital filters (Nakajima et al., 1996) and empirical mode decomposition (Garde et al., 2013) were successfully used, among others. These algorithms have mostly focused on estimating average RR from a window of PPG. For example, Karlen et al. (2013) and Garde et al. (2014) estimated RR every second using 16, 32, 64-s windows of PPG data.

Few algorithms, however, have proposed to estimate RR instantaneously (IRR), mostly performed by the time-frequency approaches based on a continuous wavelet (Clifton et al., 2007; Addison et al., 2012), variable frequency complex demodulation methods (VFCDM) (Chon et al., 2009) and short-time Fourier analysis (STFT) (Shelley et al., 2006).

In this study, we have proposed a novel method for extracting the instantaneous respiratory rate (IRR) from PPG. The method was performed in three main steps: extraction of RIIV, RIAV, and RIFV signals from PPG, estimation of IRR from each extracted respiratory-induced variation signals and fusion of IRR estimates. A time-frequency transform called synchrosqueezing transform (SST) (Daubechies et al., 2011) was used to extract RIIV, RIAV, and RIFV from PPG. Later, a second SST was applied to estimate IIR from respiratory-induced variation signals (Addison and Watson, 2004). To combine IRR estimates corresponding to each respiratory-induced variation signal, a novel method, called peak-conditioned fusion algorithm was proposed.

## 2. Algorithm description

### 2.1. Instantaneous frequency (IF)

The instantaneous frequency is the frequency at a given time. Consider a multicomponent signal *f* that can be modeled as

(1) $$\begin{array}{r} f\left(t\right)=\overset{K}{\underset{k\;=\;1}{\sum }}f_{k}\left(t\right)=\overset{K}{\underset{k\;=\;1}{\sum }}A_{k}\left(t\right)\cos\left(2\pi \phi_{k}\left(t\right)\right) \end{array}$$

where *A*_*k*_(*t*) and ϕ_*k*_(*t*) are the time-varying amplitude and phase of *kth* frequency component, respectively.

In theory, the instantaneous frequency (*IF*) is defined as the derivative of the phase function with respect to time as

(2) $$\begin{array}{r} IF_{f}={\left\{\phi_{k}^{\prime }\left(t\right)\right\}}_{1\leq k\leq K} \end{array}$$

### 2.2. Synchrosqueezing transform

Synchrosqueezing Transform (SST) was first introduced by Daubechies et al. (2011) in 1996 and then implemented by Thakur et al. (2013). SST is a combination of wavelet analysis and a reallocation method which sharpens a time-frequency representation by allocating its points to another locations in the time-frequency plane. SST can provide an accurate estimation of IF.

As defined in Daubechies et al. (2011), SST involves three steps:

**Step 1:** Estimation of the continuous wavelet transform (CWT)

The CWT of *f* is calculated as

(3) $$\begin{array}{r} W_{f}\left(a,b\right)=\int f\left(t\right)a^{-½}\bar{\psi \left(\frac{t-b}{a}\right)}\text{d}t \end{array}$$

where ψ is a wavelet with $\hat{\psi }\left(\xi \right)=0$ for ξ ≤ 0 and *a* and *b* are scale and location variables, respectively. $\bar{\psi }\left(\xi \right)$ is the complex conjugate of ψ(ξ) and $\hat{\psi }\left(\xi \right)$ is the Fourier transform of ψ(ξ) estimated as

(4) $$\begin{array}{r} \hat{\psi }\left(\xi \right)=\int \psi \left(\xi \right)e^{-i\left(2\pi \xi \right)t}\text{d}t \end{array}$$

**Step 2**: Estimation of the instantaneous frequency

If $\hat{\psi }\left(\xi \right)$ is concentrated around ξ = ω_0_, then *W*_*f*_(*a, b*) will be spread out around the horizontal line $a{=}^{\omega_{0}}{/}_{\omega }$ on the time-scale presentation for a given frequency of ω. However, Daubechies et al. (2011) showed that the oscillation of *W*_*f*_(*a, b*) around b tends to the original frequency ω, irrespective of the value of *a*. Therefore, for any (*a, b*) where *W*_*f*_(*a, b*) ≠ 0, the instantaneous frequency ω_*f*_(*a, b*) for signal *f* can be defined as

(5) $$\begin{array}{r} \omega_{f}\left(a,b\right)=-\frac{i}{2\pi }\left({\left(W_{f}\left(a,b\right)\right)}^{-1}\frac{\partial }{\partial b}W_{f}\left(a,b\right)\right) \end{array}$$

**Step 3**: Transfer to the time-frequency plane

In this step, each point on the time-scale plane is allocated to a point on the time-frequency plane using the map (*a, b*) → (ω_*f*_(*a, b*), *b*). The frequency variable ω and the scale variable *a* are both binned: *W*_*f*_(*a, b*) is computed only at discrete values *a*_*k*_, with *a*_*k*_ − *a*_*k*−1_ = (Δ*a*)_*k*_ and its SST, *T*_*f*_(ω, *b*), is estimated only at the centers ω_*l*_ of the successive bins $\left[\omega_{l}-\frac{1}{2},\omega_{l}+\frac{1}{2}\right]$, with ω_*l*_−ω_*l*−1_ = Δω, by summing different points:

(6) $$\begin{array}{r} T_{f}\left(\omega_{l},b\right)={\left(\Delta \omega \right)}^{-1}\underset{a_{k}:|\omega \left(a_{k},b\right)-\omega_{l}|\leq \frac{\Delta \omega }{2}}{\sum }W_{f}\left(a_{k},b\right)a_{k}^{\frac{-3}{2}}{\left(\Delta a\right)}_{k}. \end{array}$$

## 3. Materials and methods

### 3.1. Data set

#### 3.1.1. Capnobase data set

The Capnobase contains test and calibration data sets (Karlen et al., 2010). Test data set contains forty-two 8-min segments of recordings obtained from 29 pediatric and 13 adults receiving general anesthesia at the British Columbia Children's Hospital and St. Paul's Hospital, Vancouver, BC, respectively. Calibration data set contains one hundred twenty-four 2-min segments of recordings used for tuning the parameters of the proposed algorithm.

In both data sets, the recordings included ECG, capnometry, and PPG (sampled at 300, 300, and 100 Hz, respectively) obtained with S/5 collect software (Datex-Ohmeda, Finland). The capnography waveform was used as the reference gold standard recording for RR. A research assistant manually labeled each breath in the capnogram. The beginning and end of all artifacts in the PPG waveforms were also manually labeled. Both datasets can be downloaded from the on-line database, CapnoBase.org.

#### 3.1.2. Sleep data set

Sleep database contains forty-three 20-min segments of recording from 43 children (age = 9.1 ± 4.1, Apnea/Hypopnea Index = 8.9 ± 17.9) referred to the British Columbia Children's Hospital for overnight standard polysomnography (PSG). The children had been recruited following approval by the University of British Columbia Clinic Research Ethics Board (H11-01769) and informed parental consent. Parental/guardian written informed consent was obtained for all children. Children with a cardiac arrhythmia or abnormal hemoglobin were excluded from the study.

Standard PSG recordings included overnight measurements of ECG, electroencephalography (EEG), oxygen saturation (SpO_2_), PPG, chest and abdominal movement, nasal and oral airflow, left and right electrooculography (EOG), electromyography (EMG) and video capture. The PSG recordings were performed with the Embla Sandman S4500 (Embla Systems, ON, Canada).

In addition to PSG, the PPG was recorded simultaneously using the Phone Oximeter™ (Karlen et al., 2011) sampled at 62.5 Hz with 32-bit resolution.

For each subject, the 20 min of recordings were selected after 2 h of sleep. Apnea/hypopnea events were annotated as the artifacts and no specific sleep stage was selected.

The nasal/oral airflow waveform was used as the reference gold standard recording for RR. Two experts manually labeled each breath in nasal/oral airflow waveform. The beginning and end of all artifacts in the oral/nasal waveforms were also manually labeled.

Sleep database is available for download at https://figshare.com/s/552ec33f37ae8d99c032 (doi: 10.6084/m9.figshare.6683807).

### 3.2. Estimation of IRR from PPG

**Figure 2:**
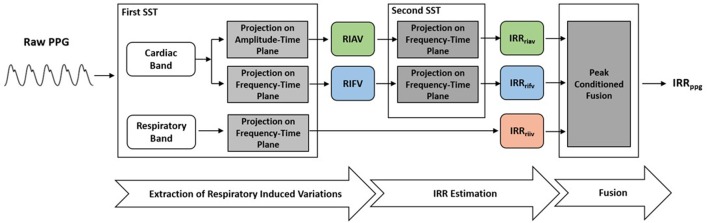
To extract IRR from PPG, the first SST was applied to PPG to extract RIIV, RIAV, and RIFV. Later, the second SST was performed to estimate IIR from a respiratory-induced variation signals. The peak-conditioned fusion algorithm was then used to fuse simultaneous IRR estimates.

**Figure 3:**
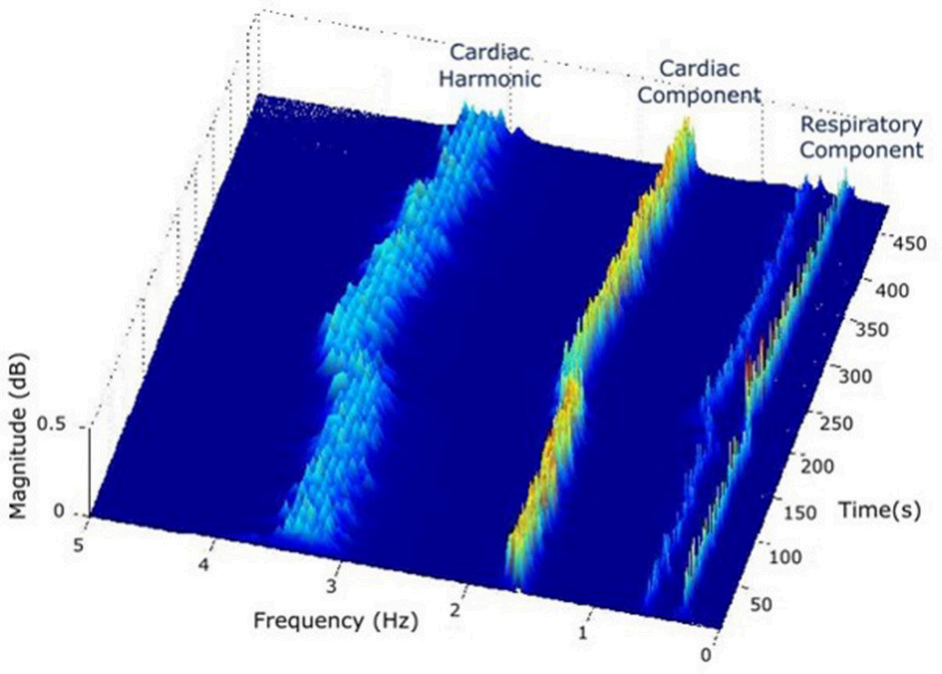
In the STT surface of PPG, two components are identified: a strong cardiac component in the cardiac band (0.5–3 Hz, 30–180 beats/min) and a respiratory component in the respiratory band (0.14–0.9 Hz, 8–54 breaths/min).

To perform IRR estimation, after a preprocessing stage, a first SST was applied to PPG to extract RIIV, RIAV and RIFV. Later, a second SST was performed to estimate IIR from a respiratory-induced variation signals. The peak-conditioned fusion algorithm was then used to fuse simultaneous IRR estimates. This procedure, inspired by the method known as secondary wavelet feature decoupling (SWFD) (Addison and Watson, 2004), involves the following steps (Figure 2):

(1) The first SST is applied to the PPG signal.
(2) In the STT surface, two components are identified: a strong cardiac component in the cardiac band (0.5–3 Hz, 30–180 beats/min) and a respiratory component in the respiratory band (0.14–0.9 Hz, 8–54 breaths/min) (Figure 3).

In this study, reference ranges of cardiac and respiratory bands were extracted from a review of observational studies that used HR from 143,346 children and RR data from 3,881 children (from 6 months to 18 years old) (Fleming et al., 2011). Based on 99th and 1st centiles for children and young adults, the HR could range from 30 to 180 beats/min (0.50 to 3 Hz, respectively) and RR from 8 to 54 breaths/min (0.14 to 0.9 Hz, respectively). The range in adults is much more restricted, thus it would be included in this range.

(3) The respiratory component in SST surface shows RIIV and its ridge in the frequency-time plane represents RIIV-derived IRR (IRR_*riiv*_) (Figure 3).
(4) The ridge of cardiac component is followed either in the amplitude-time plane to get RIAV or in the frequency-time plane to get RIFV. This is done by projecting the cardiac ridge points onto the amplitude-time or frequency-time planes, respectively.
(5) The second SST applied to RIAV results in a dominant single component in the respiratory band (0.14–0.9 Hz, 8–54 breaths/min) whose ridge represents RIAV-derived IRR (IRR_*riav*_).
(6) A second SST is applied to the RIFV signal as well to get a dominant single component in the respiratory band whose ridge represents RIFV-derived IRR (IRR_*rifv*_).
(7) Estimation of final IRR (IRR_*ppg*_) is performed using a proposed peak frequency tracking method (so-called peak-conditioning fusion) which combines the instantaneous frequency information from (IRR_*riiv*_), (IRR_*riav*_) and (IRR_*rifv*_).

#### 3.2.1. Preprocessing

The PPG signals were lowpass filtered by a lowpass Chebyshev Type I IIR filter of order 8 and down sampled to 10 Hz.

#### 3.2.2. Estimation of *IRR*_*riiv*_

Consider a PPG signal as a vector *ppg*∈*R*^*n*^, *n* = 2^*L*+1^ where *L* is a nonnegative integer. The CWT of *ppg*, *W*_*ppg*_, was calculated using the Morlet wavelet, ψ, where its Fourier transform was concentrated around 1.25 Hz. The *W*_*ppg*_ was sampled at the location (*a*_*j*_, *b*), where $a_{j}=2^{j/n_{v}}$, *j* = 1, …, *Ln*_*v*_, *n*_*v*_ = 32 and *b* = 1, …, *n*. The result is a *Ln*_*v*_×*n* matrix denoted ${\tilde{W}}_{ppg}$.

When ${\tilde{W}}_{ppg}>0$, ${\tilde{\omega }}_{ppg}$ was implemented as follow

(7) $$\begin{array}{r} {\tilde{\omega }}_{ppg}=-\frac{i}{2\pi }D_{b}{\tilde{W}}_{ppg}\left(a_{j},b\right){\tilde{W}}_{ppg}{\left(a_{j},b\right)}^{-1} \end{array}$$

where $D_{b}{\tilde{W}}_{ppg}$ was the finite differences of ${\tilde{W}}_{ppg}$ with respect to *b*.

Then frequency variable, ω, was binned into frequency division $\omega_{l}=2^{l△\omega }$ω, *l* = 0, …, *Ln*_*v*_−1, where $△\omega =\frac{1}{Ln_{v}-1}log_{2}\left(\frac{n}{2}\right)$, ω = $\frac{1}{n△t}$ and $\overset{-}{\omega }=\frac{1}{2△t}$. $\overset{-}{\omega }$ and ω, were maximum and minimum frequencies respectively and were chosen based on Nyquist sampling theorem.

The SST of PPG was calculated as

(8) $$\begin{array}{r} T_{ppg}\left(\omega_{l},b\right)=\underset{a_{j}:|\omega \left(a_{j},b\right)-\omega_{l}|\leq \frac{\Delta \omega }{2}}{\sum }\frac{log2}{Ln_{v}}{\tilde{W}}_{ppg}\left(a_{j},b\right)a_{j}^{\frac{-1}{2}}. \end{array}$$

*T*_*ppg*_ over time shows both cardiac and respiratory bands (Figure 3).

A ridge fitting the dominant area of *T*_*ppg*_ in the respiratory band (0.14–1 Hz) represented *IRR*_*riiv*_ and was extracted by tracking the local maximum values in this region.

#### 3.2.3. Estimation of *IRR*_*riav*_

Consider *RIAV* as a vector *riav*∈*R*^*n*^, where *n* is the length of *ppg*. In the amplitude-time plane of *T*_*ppg*_, *riav* estimated as a ridge fitting the dominant area of *T*_*ppg*_ in the cardiac band (0.5–3 Hz, 30–180 beats/min). The ridge extracted by finding the local maximum values which minimize the following cost function (Abid et al., 2007):

(9) $$\begin{array}{r} Cost=\overset{n}{\underset{b\;=\;1}{\sum }}\left[-|T_{ppg}\left(riav\left(b\right),b\right){|}^{2}+|riav\left(b\right)-riav\left(b-1\right){|}^{2}\right] \end{array}$$

The SST of *riav*, *T*_*riav*_ was calculated using the same implementation described in the previous section.

A ridge fitting the dominant area of *T*_*riav*_ in the respiratory band (0.14–0.9 Hz) represented the RIAV-derived IRR (*IRR*_*riav*_) and can be extracted by tracking the local maximum values in this region.

#### 3.2.4. Estimation of *IRR*_*rifv*_

Consider *RIFV* as a vector *rifv*∈*R*^*n*^, where *n* is the length of *ppg*. In the frequency-time plane of *T*_*ppg*_, *rifv* estimated as a ridge fitting the dominant area of *T*_*ppg*_ in the cardiac band (0.5–3 Hz, 30–180 beats/min). The ridge extracted by finding the local maximum values which minimize the following cost function (Abid et al., 2007):

(10) $$\begin{array}{r} Cost=\overset{n}{\underset{b\;=\;1}{\sum }}\left[-|T_{ppg}\left(rifv\left(b\right),b\right){|}^{2}+|rifv\left(b\right)-rifv\left(b-1\right){|}^{2}\right] \end{array}$$

The SST of *riav*, *T*_*riav*_ was calculated using the same implementation described in the section 3.2.2.

A ridge fitting the dominant area of *T*_*riav*_ in the respiratory band (0.14–0.9 Hz) represented the RIFV-derived IRR (*IRR*_*rifv*_) and can be extracted by tracking the local maximum values in this region.

#### 3.2.5. Peak-conditioned fusion

The peak-conditioned fusion method, inspired by Lázaro Plaza (2015), was proposed to combine the IRR estimates from three respiratory-induced variations to provide the final *IRR*_*ppg*_.

**Figure 4:**
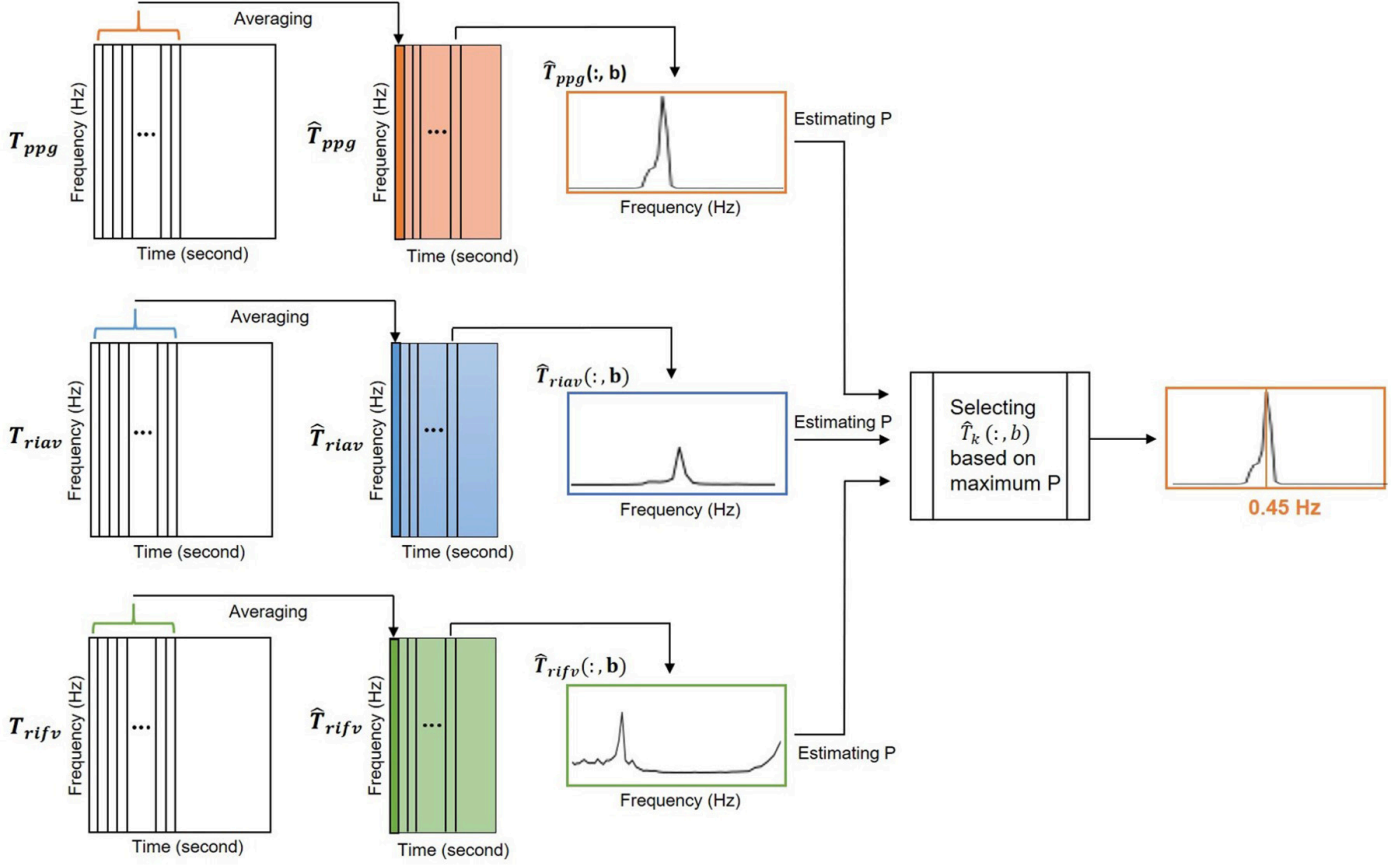
The peak-conditioned fusion method combined the IRR estimates from three respiratory-induced variations to provide the final IRR.

The calculated *T*_*ppg*_, *T*_*riav*_ and *T*_*rifv*_ are two-dimensional matrices $\in R^{Ln_{v}n}$, *n* = 2^*L*+1^ where *L* is a nonnegative integer and *n*_*v*_ = 32. Each column of *T*_*ppg*_, *T*_*riav*_ and *T*_*rifv*_ matrices shows the frequency distribution of PPG, RIAV and RIFV signals at each time instance, respectively. To reduce the variance, each matrix is averaged in time dimension using a moving window of length *T*_*m*_ = 16 s every *t*_*s*_ = 5 s. The averaged matrix is denoted as ${\hat{T}}_{k}$, where *k* refers to *ppg*, *riav*, or *rifv* (Figure 4).

At instant b, the location of the largest peak in respiratory band of each ${\hat{T}}_{k}\left(:,b\right)$ column (for k = ppg, riav or rifv) is detected and denoted as $ir_{k}^{I}\left(b\right)$. Then, a reference frequency interval, Ω_*k*_(*b*), was defined as

(11) $$\begin{array}{r} \Omega_{k}\left(b\right)=\left[f\left(b-1\right)-\delta ,f\left(b-1\right)+2\delta \right] \end{array}$$

where (*b* − *1*) was a respiratory rate reference estimated from the *b* − *1* previous step.

All peaks larger than 85% of $ir_{k}^{I}\left(b\right)$ inside Ω(*b*) were detected and $ir_{k}^{II}\left(b\right)$ was chosen as the nearest to *f*_(*b*−*1*)_. By reaching to this point, $ir_{riiv}^{II}\left(b\right)$, $ir_{riav}^{II}\left(b\right)$ and $ir_{rifv}^{II}\left(b\right)$ were available simultaneously.

The final respiratory peak at instant b, *IIR*_*ppg*_((*b*)), was then chosen among $ir_{riiv}^{II}\left(b\right)$, $ir_{riav}^{II}\left(b\right)$ and $ir_{rifv}^{II}\left(b\right)$ estimates with the largest *P*_*k*_. *P*_*k*_ is a measure of the peakness and was defined as the ratio of power contained in an interval centered around the largest peak to the power of Ω_*k*_(*b*). *P* mathematically calculated as

(12) $$\begin{array}{r} p_{k}\left(b\right)=\frac{\sum_{max\left\{if_{k}^{II}\left(b\right)-0.6\delta ,f\left(b\right)-\delta \right\}}^{min\left\{if_{k}^{II}\left(b\right)+0.6\delta ,f\left(b\right)+2\delta \right\}}\hat{T_{k}\left(:,b\right)}}{\sum_{f\left(b\right)-\delta }^{f\left(b\right)+2\delta }\hat{T_{k}}\left(:,b\right)} \end{array}$$

Estimation of respiratory rate as the largest peak in the respiratory band would increase the risk of choosing the location of false peaks. To decrease this risk, the search for the largest peak was limited to the reference frequency interval, Ω_*k*_(*b*) (Lázaro Plaza, 2015). This is an asymmetric interval of 3δ centered around a reference frequency. At each step the respiratory rate reference was updated using

(13) $$\begin{array}{r} f\left(b+1\right)=\beta *f\left(b\right)+\left(1-\beta \right)*IRR_{ppg}\left(b\right) \end{array}$$

where $f\left(b\right)=arg\;max\left({\hat{T}}_{k}\left(:,1\right)\right)$ in the frequency band of [*0*.*2Hz, 0*.*7Hz*].

Value of δ was set as 0.1 and the value of *a* was tuned as 0.6 over the calibration data set.

### 3.3. Algorithm evaluation

To evaluate the performance of SST-based algorithms, agreement between reference IRR and estimated IRR (using peak-conditioned fusion, simple fusion, single respiratory-induce variation) was assessed using the limits of agreement (LOA) technique. The bias and 95% LOA were estimated using the Bland-Altman plot. Since for each subject multiple measurement were observed, the Bland-Altman method for multiple observations per individual (Zou, 2013) was used instead of the standard Bland-Altman method. The bias was calculated as mean of IRR_*est*_ - IRR_*ref*_ and the 95% LOAs as mean bias ± 1.95 standard deviations. Two standard deviations (2SD) were also estimated in the purpose of ranking the proposed algorithm in this study based on the statistical analysis reported by Charlton et al. (2016).

The coverage probability (CP_2_) was also reported as the probability of measurement error falling within pre-defined bounds, set as 2 breaths per minute (bpm) in this study (Barnhart et al., 2007).

In addition, the performance of algorithms was assessed using the root mean square error (RMSE) (breaths/min) defined as

(14) $$\begin{array}{r} RMSE=\sqrt{\frac{1}{n}\overset{n}{\underset{i\;=\;1}{\sum }}{\left(IRR_{ref}-IRR_{est}\right)}^{2}} \end{array}$$

where *n* is the number of observations and IRR_*ref*_ and IRR_*est*_ are the reference and the algorithm estimates, respectively.

## 4. Results

### 4.1. Capnobase data base

IRR extracted from the capnography waveform (IRR_*CO*2_) was used as the reference gold standard. The distribution of the respiratory rates contained 3,542 data points estimated every 5 s from IRR_*CO*2_ for the 16 s moving windows over the whole dataset. The respiratory rates ranged from the lowest value of 3.6521 bpm to the highest value of 44.22 bpm. The mean rate was 15.02 bpm with standard deviation of 7.66 bpm. About 7.7% of the data points were excluded from the further analysis due to to poor signal quality of the capnography signals.

**Figure 5:**
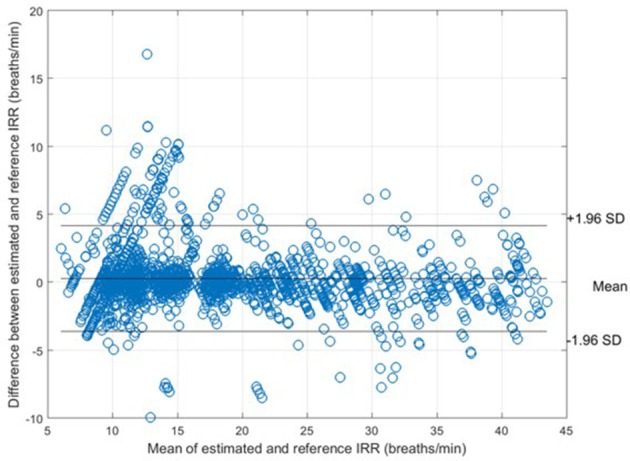
Bland-Altman plot for comparison of IRR_*CO*2_ to IRR_*ref*_ for all subjects. The bias and 95% LOAs are shown as solid lines. The bias was 0.28 and the limits of agreement −3.62 to 4.17.

For each algorithm, the measures of agreement between the estimated IRR from PPG (IRR_*est*_) and IRR_*CO*2_ and also RMSE were estimated (Table 1). For peak selection algorithm, bias was estimated as 0.28 bpm with the 95% LOAs from −3.62 to 4.17 (Figure 5). The value of 2SD was estimated as 3.97 bpm. The value of CP_2_ showed that for 89% of the IRR estimates, the measured error was less than 2 breaths/min. RMSE was estimated as 1.8 bpm.

**Table 1:** The performance of different method for estimation IRR from PPG.

	**Different IRR estimation method**	**2SD**	**Bias**	**95% LOA**	**CP_2_**	**Proportion of windows with IRR estimate (%)**	**RMSE**
	RIIV	8.8	0.3	−8.3 to 9.0	88	100	4.1
	RIAV	16.0	1.3	−14.5 to 16.9	60	100	7.5
Capnobase dataset	RIFV	9.2	0.04	−9.0 to 9.1	74	100	4.2
	Simple Fusion	8.3	0.5	−7.6 to 8.7	63	100	4.0
	Peak-Conditioned Fusion	4.0	0.3	−3.6 to 4.2	89	100	1.8
	RIIV	11.0	0.7	−10.1 to 11.4	80	100	5.1
	RIAV	21.3	5.6	−15.4 to 26.5	31	100	11.3
Sleep dataset	RIFV	8.4	−0.1	−8.4 to 8.2	79	100	3.7
	Simple Fusion	9.5	2.0	−7.3 to 11.3	41	100	4.6
	Peak-Conditioned Fusion	5.9	0.04	−5.7 to 5.8	85	100	2.3

The values of 2SD of the other algorithms ranged from 8.32 bpm to 16.00 bpm.

### 4.2. Sleep database

IRR extracted from the nasal/oral airflow waveform (IRR_*nas*_) was used as the reference gold standard in the sleep dataset. The distribution of the respiratory rates contained 10,553 data points estimated every 5 s from IRR_*nas*_ over the 16 s moving window for all subjects. The respiratory rates ranged from the lowest value of 9.561 bpm to the highest value of 50.85 bpm. The mean rate was 18.64 bpm with standard deviation of 5.66 bpm. About 0.66% of the data points were excluded from the further analysis due to to poor signal quality of the nasal/oral airflow signals.

**Figure 6:**
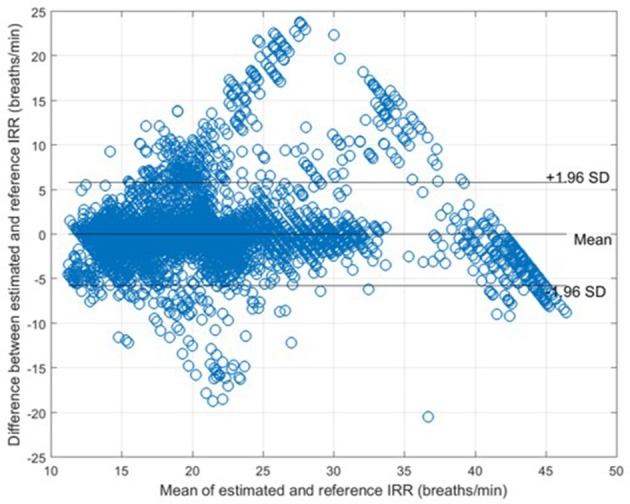
Bland-Altman plot for comparison of IRR_*nas*_ to IRR_*ref*_ for all subjects. The bias and 95% LOAs are shown as solid lines. The bias was 0.04 and the limits of agreement −5.74 to 5.82.

The measures of agreement between the estimated IRR from PPG (IRR_*est*_) and IRR_*nas*_ also RMSE were estimated for each algorithm (Table 1). For peak selection algorithm, bias was estimated as 0.04 bpm with the 95% LOAs from −5.74 to 5.82 (Figure 6). The value of 2SD was estimated as 5.90 bpm. The value of CP_2_ showed that for 85% of the IRR estimates, the measured error was less than 2 breaths/min. RMSE was estimated as 2.3 bpm.

The values of 2SD of the other algorithms ranged from 8.4 bpm to 21.3 bpm.

## 5. Discussion and conclusion

In this study, we presented an algorithm to extract IRR from PPG. We extracted RIIV, RIAV, and RIFV from PPG using Synchrosqueezing Transform (SST), a sharpening time-frequency method which provides instantaneous frequency rate. The peak-conditioned fusion was proposed to combine the extracted information from three respiratory induced variations waveforms to estimate respiratory rate at each instance. We validated the implemented method with capnography and nasal/oral airflow as the reference RR. Compared to simple fusion and single respiratory-induced variation estimations, peak-conditioned fusion shows better performance (Table 1). It provided a bias of 0.28 bpm with the 95% LOAs ranging from −3.62 to 4.17, validated against capnography (in the Capnobase dataset) (Figure 5) and a bias of 0.04 bpm with the 95% LOAs ranging from −5.74 to 5.82, validated against nasal/oral airflow (in the Sleep dataset) (Figure 6).

In this study, the proposed method estimated IRR from three sources of respiratory-induced variation and fused the estimated rates to measure the final IRR. Our findings showed that fusion of estimation rates would increase the accuracy and robustness of RR estimation. Even the simple fusion compared to single respiratory-induced variation estimations showed higher rank (narrower 2SD and greater CP2). It is consistent with the findings of Li et al. (2010) that respiratory activity may induce variation in PPG differently in different individuals. As discussed by Karlen et al. (2013), ventilatory conditions (spontaneous or mechanical ventilation) can change the behavior of respiratory induced variations.

In this study, we applied the proposed algorithm to two different data sets to include a broad range of subjects into the study. The Capnobase data set includes children adults, under controlled ventilation or spontaneously breathing over a wide RR range. The subjects were under general anesthesia and were continuously monitored. The sleep dataset includes children from 1-month to 17 years old spontaneously breathing during the overnight sleep in a sleep lab. During the overnight recordings, respiratory rates might change significantly during different sleep stages (light sleep, deep sleep or rapid eye movement (REM) sleep). In addition, some of the children may have experienced periods of breathing cessation, or obstructive sleep apnea. Since the apnea/hypopnea events induce changes on the morphology of nasal/oral airflow, i.e., flat line during apnea or very fast and low-amplitude oscillations during hypopnea, to extract the accurate reference RR from the nasal/oral airflow waveform, we labeled the apnea/hypopnea events as the artifact on nasal/oral airflow waveform.

A recent study (Charlton et al., 2016) presented a very complete assessment of RR estimation using PPG. A wide range of available techniques for estimation of respiratory-induced variations from PPG, estimation of RR from respiratory-induced variations, and fusion of RR estimates were identified and then more than 300 algorithms were implemented by assembling all possible combinations of available techniques. The algorithms were applied to the Vortal data set of 39 resting adults, with RR ranging between 5 and 32 bpm and ranked based on 2SD. The first ten top-ranked algorithms had the 2SD values ranging from 6.2 to 7.9. To be able to compare the performance of our algorithm with the ten top-ranked algorithms, we applied our proposed method on the same data set. For peak conditioned algorithm, the value of 2SD was estimated as 6.83 bpm. It ranked our algorithm among the top ten algorithms; however, the performance of our algorithm slightly decreased when RR was lower than 8 breaths/min. In addition, for the top ranked algorithms, the value of CP_2_ was reported as 71.5 while we obtained a CP_2_ of 75 applying our proposed algorithm.

It is important to note that all top ranked algorithm reported in Charlton et al. (2016) estimated RR using 32-s windows while our method can estimate RR instantaneously. It suggests that our algorithm shows better performance compared to methods that extract IRR based on time-frequency analysis (Li et al., 2010; Addison et al., 2012).

In Charlton et al. (2016), the methods for extracting RR from ECG were assessed as well. The findings of that study showed that algorithms performed better when using ECG than PPG. The best algorithm had 95% LOAs of −4.7 to 4.7 bpm and a bias of 0.0 bpm when using the ECG.

In Charlton et al. (2016), the performance of thoracic Impedance Pneumography (IP) were assessed as well providing a bias of −0.2 bpm with 95% LOAs of −5.6 to 5.2 bpm. Thoracic IP is a commonly-used technique for continuous monitoring of RR that measures changes in the electrical impedance of the person's chest during respiration. Our results showed that the performance of our algorithm is comparable with the performance of thoracic IP.

Several studies based on the continuous wavelet transform (CWT) (Clifton et al., 2007; Addison et al., 2012), the short-time Fourier transform (STFT) (Shelley et al., 2006), and empirical mode decomposition (EMD) (Garde et al., 2014) have been proposed to detect RR from PPG. The results of a study conducted by Thakur et al. (2013) to compare SST to CWT, STFT and EMD showed the superior precision of SST at identifying components of complicated oscillatory signals. Moreover, the study showed that time-varying instantaneous frequencies could be clearly distinguished in the SST while there is much more smearing and distortion in the CWT and STFT.

In this study, the SST algorithm has been implemented with O(nlog(n)) computations per scale and peak-conditioned fusion algorithm has been implemented with O(n) computations where n is the number of samples. However, there are some fast algorithms to reduce the number of computations of SST implementation to O(n) per scale Rioul and Duhamel (1992).

This study introduces a new method to estimate IRR from pulse oximetry. This would expand the functionality of a conventional pulse oximetry beyond the measurement of HR and SpO_2_ to measure the respiratory rate continuously and instantly in the clinical setting and at home. Importantly, these are all achievable with a simple, cheap, single-sensor solution.

## Author contributions

All authors made substantial contributions to the conception and design of the paper. PD contributed to data acquisition and processed the obtained data, designed, developed and evaluated the algorithm, and drafted the review. AG contributed to data acquisition and the design of algorithm and revised the paper critically for content. BM contributed to the design of algorithm and revised the paper critically for content. JA and GD revised the paper critically for content. AG and BM have contributed equally to this work. All authors approved the final submission of the document and agreed to be accountable for all aspects of the work.

### Conflict of interest statement

JA and GD are founders of LGT Medical Inc. and retain equity in the company that is commercializing a newer version of the Phone Oximeter™ used in this paper, unrelated to the manuscript. BM is employed by LGT Medical Inc. The remaining authors declare that the research was conducted in the absence of any commercial or financial relationships that could be construed as a potential conflict of interest.
